# Supplementary material for: Hot spot prediction in protein-protein interactions by an ensemble system
Source: BMC Syst Biol. 2018 Dec 31;12(Suppl 9):132. doi: 10.1186/s12918-018-0665-8 (PMC6311905; doi:10.1186/s12918-018-0665-8)
Supplement: Supplementary file 2 — All relevant MATLAB code. A simple Matlab implement of our predictor. (ZIP 19,742 kb) [file 12918_2018_665_MOESM2_ESM.zip › Additional file 2/Read ASA/ASA website/SKEMPI/1XD3B.htm]

 NetSurfP - Protein Surface Accessibility and Secondary Structure Predictions

  

|  |  |  |
| --- | --- | --- |
|  |  | NetSurfP - Protein Surface Accessibility and Secondary Structure Predictions  Technical University of Denmark |

  


---

```
# For publication of results, please cite:
# A generic method for assignment of reliability scores applied to solvent accessibility predictions.
# Bent Petersen, Thomas Nordahl Petersen, Pernille Andersen, Morten Nielsen and Claus Lundegaard
# BMC Structural Biology 2009, 9:51 doi:10.1186/1472-6807-9-51
#
# Column 1: Class assignment - B for buried or E for Exposed - Threshold: 25% exposure, but not based on RSA
# Column 2: Amino acid
# Column 3: Sequence name
# Column 4: Amino acid number
# Column 5: Relative Surface Accessibility - RSA
# Column 6: Absolute Surface Accessibility
# Column 7: Z-fit score for RSA prediction
# Column 8: Probability for Alpha-Helix
# Column 9: Probability for Beta-strand
# Column 10: Probability for Coil
E M  1XD3B                  1    0.370  74.017  -0.966   0.010   0.072   0.918
E Q  1XD3B                  2    0.422  75.423   2.020   0.001   0.959   0.040
B I  1XD3B                  3    0.038   7.104  -0.197   0.000   0.983   0.017
B F  1XD3B                  4    0.217  43.512   1.605   0.000   0.983   0.017
B V  1XD3B                  5    0.045   6.932   0.468   0.000   0.983   0.017
E K  1XD3B                  6    0.276  56.794   1.281   0.000   0.983   0.017
B T  1XD3B                  7    0.136  18.808   0.564   0.002   0.816   0.182
E L  1XD3B                  8    0.315  57.695  -0.045   0.005   0.262   0.733
E T  1XD3B                  9    0.692  95.980   0.333   0.005   0.015   0.979
E G  1XD3B                 10    0.668  52.595  -0.837   0.003   0.003   0.994
E K  1XD3B                 11    0.551 113.300   0.667   0.005   0.262   0.733
E T  1XD3B                 12    0.521  72.249   1.373   0.001   0.900   0.099
B I  1XD3B                 13    0.124  23.014   1.776   0.001   0.959   0.040
E T  1XD3B                 14    0.402  55.730   1.507   0.001   0.959   0.040
B L  1XD3B                 15    0.044   8.056   0.782   0.001   0.959   0.040
E E  1XD3B                 16    0.504  88.119   1.248   0.001   0.959   0.040
B V  1XD3B                 17    0.033   5.011   0.249   0.002   0.816   0.182
E E  1XD3B                 18    0.446  77.881   1.253   0.005   0.336   0.660
E P  1XD3B                 19    0.431  61.102  -0.668   0.018   0.047   0.935
E S  1XD3B                 20    0.658  77.118  -0.097   0.058   0.017   0.925
E D  1XD3B                 21    0.241  34.656   0.162   0.115   0.016   0.868
E T  1XD3B                 22    0.251  34.800   1.275   0.522   0.016   0.462
B I  1XD3B                 23    0.033   6.142  -0.079   0.970   0.001   0.030
E E  1XD3B                 24    0.377  65.827   1.502   0.970   0.001   0.030
E N  1XD3B                 25    0.463  67.725   1.985   0.970   0.001   0.030
B V  1XD3B                 26    0.026   3.919   1.059   0.970   0.001   0.030
B K  1XD3B                 27    0.162  33.303   1.104   0.970   0.001   0.030
E A  1XD3B                 28    0.508  55.993   1.653   0.988   0.000   0.012
E K  1XD3B                 29    0.262  53.914   2.295   0.988   0.000   0.012
B I  1XD3B                 30    0.029   5.291   0.343   0.970   0.001   0.030
E Q  1XD3B                 31    0.334  59.563   0.646   0.970   0.001   0.030
E D  1XD3B                 32    0.724 104.314   0.850   0.923   0.002   0.076
E K  1XD3B                 33    0.432  88.760   0.478   0.782   0.003   0.216
E E  1XD3B                 34    0.483  84.345   0.098   0.246   0.004   0.750
E G  1XD3B                 35    0.819  64.471   0.277   0.003   0.003   0.994
B I  1XD3B                 36    0.073  13.486   0.594   0.005   0.015   0.979
E P  1XD3B                 37    0.397  56.320   0.667   0.005   0.015   0.979
B P  1XD3B                 38    0.252  35.773  -0.831   0.053   0.043   0.903
E D  1XD3B                 39    0.668  96.201  -0.541   0.052   0.084   0.864
E Q  1XD3B                 40    0.355  63.421   0.419   0.019   0.141   0.840
B Q  1XD3B                 41    0.073  13.074   0.315   0.022   0.552   0.426
B R  1XD3B                 42    0.232  53.105   1.447   0.011   0.918   0.071
B L  1XD3B                 43    0.029   5.347   0.793   0.011   0.918   0.071
B I  1XD3B                 44    0.055  10.101   0.283   0.011   0.918   0.071
B F  1XD3B                 45    0.074  14.832  -0.261   0.018   0.846   0.136
E A  1XD3B                 46    0.305  33.666  -0.472   0.004   0.420   0.576
B G  1XD3B                 47    0.351  27.584  -1.440   0.004   0.197   0.799
E K  1XD3B                 48    0.306  62.882   0.731   0.004   0.616   0.381
E Q  1XD3B                 49    0.383  68.457   0.485   0.003   0.718   0.279
B L  1XD3B                 50    0.054   9.869   0.328   0.004   0.420   0.576
E E  1XD3B                 51    0.515  89.970   0.734   0.004   0.138   0.858
E D  1XD3B                 52    0.507  73.059  -0.216   0.018   0.047   0.935
E G  1XD3B                 53    0.701  55.161   0.013   0.058   0.017   0.925
E R  1XD3B                 54    0.432  99.020   0.874   0.113   0.043   0.844
E T  1XD3B                 55    0.241  33.482   1.508   0.199   0.152   0.649
B L  1XD3B                 56    0.068  12.414  -0.088   0.321   0.252   0.427
E S  1XD3B                 57    0.480  56.209   0.905   0.453   0.248   0.299
E D  1XD3B                 58    0.587  84.515   0.305   0.307   0.165   0.527
B Y  1XD3B                 59    0.142  30.367   1.357   0.118   0.150   0.732
E N  1XD3B                 60    0.590  86.420   0.399   0.018   0.047   0.935
B I  1XD3B                 61    0.057  10.600   0.378   0.005   0.045   0.951
E Q  1XD3B                 62    0.508  90.693   1.401   0.005   0.015   0.979
E K  1XD3B                 63    0.783 161.125   0.958   0.005   0.015   0.979
E E  1XD3B                 64    0.591 103.230  -0.312   0.005   0.015   0.979
B S  1XD3B                 65    0.109  12.787  -0.492   0.004   0.085   0.910
B T  1XD3B                 66    0.193  26.783   1.180   0.011   0.918   0.071
B L  1XD3B                 67    0.026   4.724   1.148   0.006   0.962   0.032
B H  1XD3B                 68    0.146  26.466   1.835   0.006   0.962   0.032
B L  1XD3B                 69    0.023   4.211   1.030   0.001   0.959   0.040
B V  1XD3B                 70    0.060   9.283   0.858   0.000   0.983   0.017
B L  1XD3B                 71    0.067  12.286  -0.364   0.001   0.959   0.040
B R  1XD3B                 72    0.274  62.677   0.502   0.001   0.900   0.099
B L  1XD3B                 73    0.173  31.695  -1.193   0.004   0.616   0.381
E R  1XD3B                 74    0.438 100.416   0.389   0.018   0.088   0.893
E G  1XD3B                 75    0.890  70.067  -2.153   0.000   0.000   0.999

Explain the output.  Go back.
```

---
